# Buffering roles of (pro)renin receptor in starvation‐induced autophagy of skeletal muscles

**DOI:** 10.14814/phy2.13587

**Published:** 2018-02-27

**Authors:** Yuki Mizuguchi, Midori Yatabe, Noriko Morishima, Satoshi Morimoto, Atsuhiro Ichihara

**Affiliations:** ^1^ Department of Medicine II, Endocrinology and Hypertension Tokyo Women's Medical University Tokyo Japan

**Keywords:** C2C12 cells, skeletal muscles, transcription factor EB

## Abstract

Autophagy is an intracellular catabolic process contributing to the regulation of nutrient homeostasis and cellular remodeling. Studies revealed that the nuclear translocation of transcription factor EB (TFEB) plays a key role in lysosomal biogenesis and autophagic pathways. The (pro)renin receptor [(P)RR] is a multifunctional protein playing a pivotal role in regulation of the tissue renin–angiotensin system and is known as an essential constituent of vacuolar H^+^‐ATPase, considered to be necessary for the autophagy–lysosome pathway. On the basis of these findings, we postulated that (P)RR may also contribute to the regulation of starvation‐induced autophagy. In this study, starvation increased the expression of (P)RR and autophagy‐related genes, especially, in the skeletal muscles of mice. In C2C12 mouse myoblast cells, starvation increased (P)RR expression and TFEB translocation, leading to the expression of autophagy‐related genes. Knockdown of (P)RR enhanced both the TFEB translocation to the nucleus and the expression of autophagy‐related genes during starvation. These results suggest that (P)RR plays a buffering role in starvation‐induced autophagy by affecting the nuclear translocation of TFEB. Thus, (P)RR, which increases during starvation, is one of the important factors that control autophagy in the skeletal muscles. (P)RR may act as a buffer to reduce excessive TFEB‐dependent autophagy flux.

## Introduction

Autophagy is an intracellular catabolic process contributing to the regulation of nutrient homeostasis and cellular remodeling. Studies revealed that subcellular localization of transcription factor EB (TFEB) plays a key role in lysosomal biogenesis and autophagic pathways (Pena‐Llopis et al. 2011; Settembre et al. 2011; Roczniak‐Ferguson et al. 2012). Under normal nutrient conditions, phosphorylated TFEB is retained in the cytoplasm. Cellular stress, such as nutrient starvation, induces the translocation of TFEB to the nucleus, which leads to the transcription of autophagy‐related genes and controls the major steps in autophagic pathways (Khambu et al. 2011; Settembre et al. 2012).

The (pro)renin receptor [(P)RR], the product of *ATP6AP2* gene, is a multi‐functional protein consisting of 350 amino acids with a single transmembrane domain, and it binds preferentially to renin and prorenin (Nguyen et al. 2002). The binding of prorenin to extracellular domain of the (P)RR causes nonproteolytic prorenin activation (Suzuki et al. 2003), which accelerates the conversion of angiotensinogen to angiotensin I. This process plays a pivotal role in the regulation of the tissue renin–angiotensin system (RAS) (Nguyen et al. 2002). The full‐length (P)RR is cleaved by furin to generate soluble (P)RR [s(P)RR], which is secreted into the extracellular space (Biswas et al. 2010; Yoshikawa et al. 2011). Studies have reported the usefulness of s(P)RR measurement as a method to evaluate tissue expression of (P)RR and tissue RAS activity (Watanabe et al. 2012; Hamada et al. 2013; Morimoto et al. 2014; Nishijima et al. 2014).

Previous studies, including ours, reported that the total suppression of (P)RR leads to the dysfunction of lysosomal–autophagy system in vivo and in vitro (Cruciat et al. 2010; Kinouchi et al. 2010; Oshima et al. 2011). Since (P)RR is an essential constituent of vacuolar H^+^‐ATPase (v‐ATPase), the knockout of (P)RR causes an impairment of v‐ATPase, and thus compromises acidification of autophagosome, which is necessary for the autophagy–lysosome pathway.

While high glucose is reported to upregulate (P)RR in podocytes to active PI3K/Akt/mTOR signaling and reduce autophagic flux (Li and Siragy 2015), involvement of (P)RR in starvation‐induced autophagy is not clear. Furthermore, to our knowledge, the expression of (P)RR during nutrient starvation has not been examined. Therefore, in this study, we assessed the expression of (P)RR during starvation in skeletal muscles and examined its role in starvation‐induced autophagy.

## Materials and Methods

### Animal models

All procedures and animal care were approved by our Institutional Animal Research Committee and conformed to the animal care Guideline for the Care and Use of Laboratory Animals of Tokyo Women's Medical University. Male C57BL/6J mice (10‐week‐old, 20–22 g body weight) were obtained from Tokyo Women's Medical University Laboratory Animal Center and were fed with a normal diet (CE‐2; CLEA Japan, Tokyo, Japan) containing 4.2% fat and 54.6% carbohydrate. To induce starvation, mice were fasted for 48 h with free access to tap water until sample acquisition.

### Analysis for serum s(P)RR levels

Serum concentration of s(P)RR in mice was measured by an ELISA kit (IBL, Fujioka, Japan) consisting of a solid‐phase sandwich ELISA with antibodies highly specific for s(P)RR (Maruyama et al. 2013).

### Real‐time PCR analysis

Total RNA was isolated from tissues using TRIzol^®^ reagent (Thermo Fisher Scientific, Lafayette, CO) or from cells using RNeasy column (Qiagen, Germantown, MD). Reverse transcription was performed using TaqMan reverse transcription reagents (Thermo Fisher Scientific, Lafayette, CO). Specific primers and probes for mouse (P)RR/ATP6ap2, Furin, *TFEB*,* ULK1*,* LCB3*,* ATG12*,* Sirt1*,* v‐ATPase* V1 subunit, *β*‐actin, and 18s rRNA subunit were purchased from Thermo Fisher Scientific (Lafayette, CO). mRNA expression was quantified by real‐time reverse transcription PCR (qRT‐PCR) using the ABI 7700 Sequence Detection System (Thermo Fisher Scientific, Lafayette, CO). Fold changes in gene expression were determined using the ΔΔ*C*_t_ method after normalization to *β*‐actin or 18s rRNA.

### Western blot analysis

Tissue and cell samples were solubilized in RIPA buffer with freshly added protease inhibitors (Sigma, St. Louis, MO). 10–50 micrograms of protein was loaded on any kD™ Mini‐PROTEIN TGX gels (Hercules, CA) transferred to PVDF membranes, and analyzed by western blotting using the ECL method. Molecular weight was calibrated using Precision Plus Protein Dual Color Standards (Bio‐Rad). Nuclear/cytosolic fractions were isolated using Lysopure ™ Nuclear and Cytoplasmic Extractor Kit (WAKO Chemical, Osaka, Japan), according to the manufacturer's protocol. Protein levels were quantified by ImageJ software (National Institutes of Health, Bethesda, MD).

### Antibodies

Following antibodies were used for western blot: (P)RR/ATP6ap2 (AF5716, R&D, Minneapolis, MN for mouse tissues and ab40790, Abcam, UK for C2C12 cells), TFEB (Bethyl Laboratories, Montgomery, TX), LC3B, GAPDH, *β*‐actin, histone 3 (Cell Signaling Technology, Danvers, MA), tubulin, antimouse‐HRP (Santacruz, CA), antirabbit‐HRP and antigoat HRP (R&D, Mineneapolis, MN).

### Cell culture

C2C12 mouse myoblast cells were obtained from Riken Cell Bank, and were cultured in Dulbecco's modified Eagle's medium (DMEM, Thermo Fisher Scientific, USA), supplemented with 10% fetal bovine serum (FBS; Bio‐west, Nuaille, France), 3 g/L glucose and 1% antibiotic and antimycotic solution (Nacalai Tesque, Kyoto, Japan). Cells were grown at 37°C with 5% CO_2_ air. The culture medium was changed every second day. To induce starvation, cells were grown to 80% confluence, washed with PBS and incubated with FBS‐free medium for 10–15 h unless otherwise specified. Medium containing pyruvate and glutamine was used unless otherwise specified. E‐64d protease inhibitor (Sigma‐Aldrich, 10 mg/mL for 1 h) and pepstatin A (Sigma‐Aldrich, 10 mg/mL for 1 h) were used to inhibit the lysosomal degradation of LC3B in the experiment illustrated in Figure 4D.

### Transfection of small interfering RNA

Small interfering (si) RNA oligonucleotides specific to (P)RR (MSS230245) and TFEB (MSS238272) were obtained from Invitrogen. Lipofectamine™ RNAiMax (Thermo Fisher Scientific, Lafayette, CO) was used to transfect siRNAs into C2C12 cells, according to the manufacturer's protocol. Expression of the target genes was analyzed by qRT‐PCR.

### Plasmids and cell transfection of TFEB

Mouse TFEB cDNA (MR223016) was purchased from Origene. This cDNA was subcloned into pLVSIN‐AcGFP1‐C1 (Takara, Shiga, Japan) via XhoI restriction enzyme site using In‐Fusion Cloning kit (Takara).

C2C12 cells were transiently transfected with plasmid DNA using XtremeGENE HP (Roche, Switzerland). Expression of the target gene was analyzed by qRT‐PCR.

### Statistical analysis

Data are expressed as mean ± standard deviation (SD). Differences between two groups were tested using Student's *t*‐test and between multiple groups using ANOVA. Analyses were performed using GraphPad Prism Version 6 (GraphPad software, La Jolla, CA) statistical program. A value of *P* < 0.05 was considered statistically significant.

## Results

### Expression of (P)RR and autophagy‐related genes in the skeletal muscles of fasted mice

**Figure 1:**
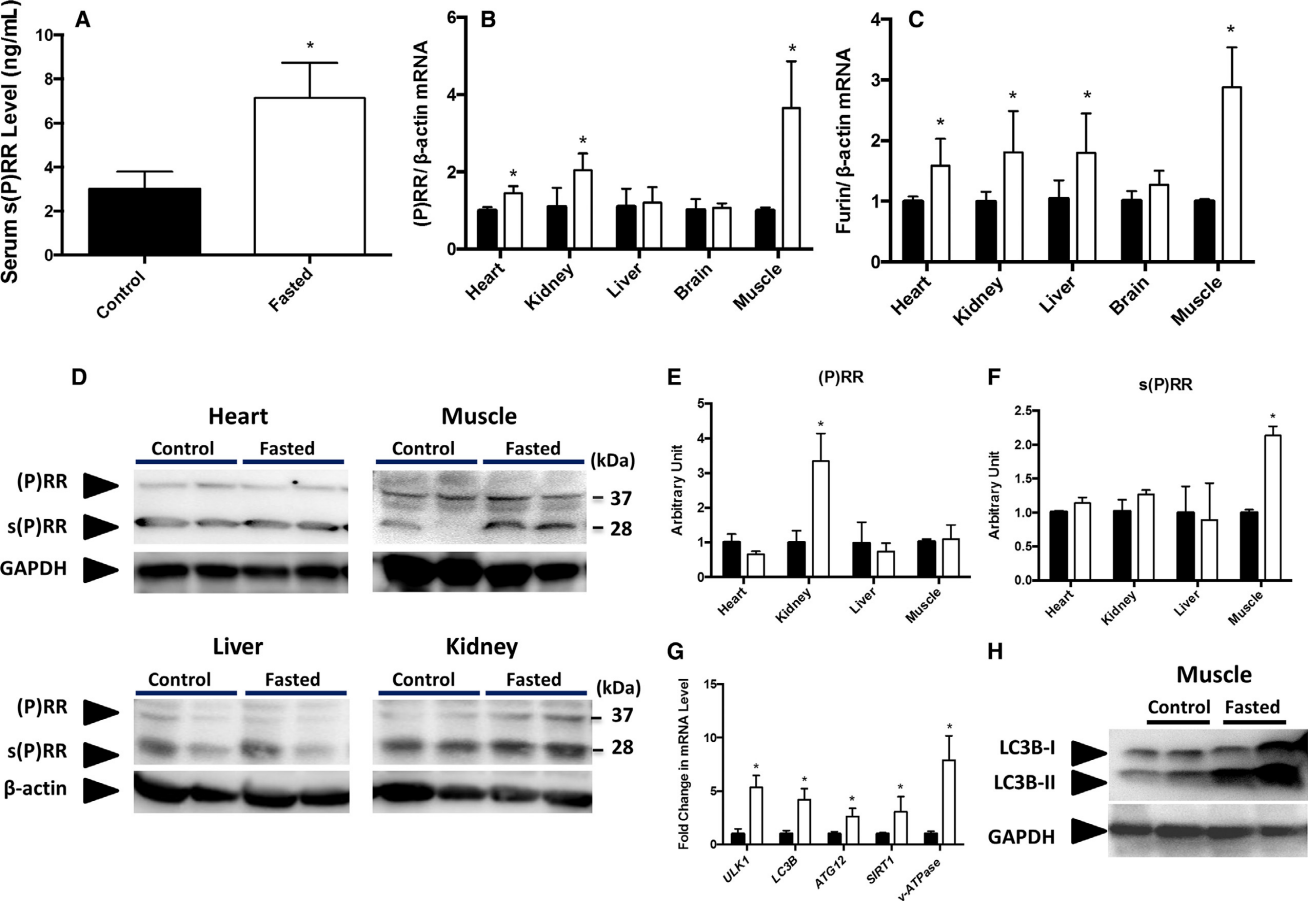
Serum s(P)RR concentration in the fasted (open square, *n* = 8) and control (closed square, *n* = 8) C57BL6 mice (A). The mRNA expression of (P)RR (B) and furin (C) in the heart, kidneys, liver, brain, and skeletal muscles of the fasted (open square, *n* = 8) and control (closed square, *n* = 6) mice. Representative western blots (D) and their quantitative analyses for (P)RR (E) and s(P)RR (F) in the heart, skeletal muscles, liver, and kidneys of the fasted and control mice. The full‐length (P)RR was detected as a 37‐kDa band and the s(P)RR was detected as a 28‐kDA band. The mRNA expression of autophagy‐related genes (*ULK1*,*LCB3*,*ATG12*), *SIRT1*, and v‐ATPase in the skeletal muscle of the fasted (open square, *n* = 10) and control (closed square, *n* = 10) mice (G). Representative western blots of LC3B in the skeletal muscle of control and fasted mice (H). Data are expressed as mean ± SD. *P < 0.05 vs. control. Muscle, skeletal muscle; (P)RR, (pro) renin receptor; s(P)RR, soluble (pro) renin receptor; *ULK1*, Serine/threonine–protein kinase ULK1; *LC3B*, microtubule‐associated protein light chain 3B; *ATG 12*, autophagy‐related protein 12; *SIRT1*, sirtuin‐1; *v‐ATPase* vacacuolar H^+^‐ATPase.

In the fasted mice, serum s(P)RR levels (7.14 ± 0.57 ng/mL) were significantly higher than those in the control mice (3.01 ± 0.56 ng/mL, Fig. 1A). Expression of (P)RR mRNA in the heart, kidneys, and skeletal muscles of the fasted mice was significantly higher than that in these organs of the control mice (Fig. 1B). The furin mRNA level was significantly higher in the heart, kidneys, liver, and especially skeletal muscles of the fasted mice than that in these organs of the control mice (Fig. 1C). Western blot analysis showed that full‐length (P)RR and s(P)RR protein levels were significantly increased in the kidneys (Fig. 1D and E) and skeletal muscles (Fig. 1D and F), respectively, of the fasted mice. Protein levels of (P)RR and s(P)RR were not augmented in the heart despite increased (P)RR mRNA, possibly due to altered protein turnover. The mRNA expression of autophagy‐related genes, *ULK1*,* LC3B*,* ATG12*,* SIRT1*, and v‐ATPase, were significantly increased in the skeletal muscles of the fasted mice as compared to that of the control mice (Fig. 1G). The expression of LC3B‐II, which is converted from LC3B‐I and used as an autophagy marker, was increased by fasting in the skeletal muscle (Fig. 1H).

### Expression of (P)RR and autophagy‐related genes in starved C2C12 cells

**Figure 2:**
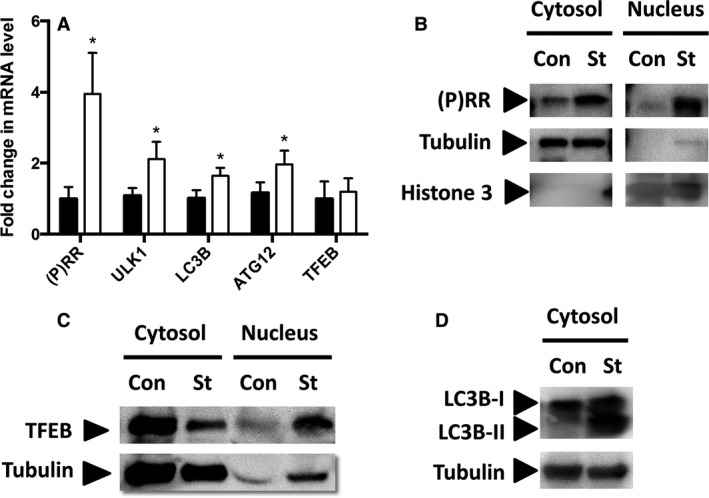
The mRNA expression of (P)RR,*ULK1*,*LC3B*,*ATG12*, and *TFEB* in the starved (open square, *n* = 6) and control (closed square, *n* = 6) C2C12 cells (A). Data are expressed as mean ± SD. *P < 0.05 vs. control. (B) Representative western blots for full‐length (P)RR in the starved and control C2C12 cells (B). Representative western blots for TFEB in the cytosol and nucleus of the starved and control C2C12 cells (C). Representative western blots for LC3B in the starved and control C2C12 cells (D). (P)RR, (pro) renin receptor; *ULK1*, Serine/threonine–protein kinase ULK1; *LC3B*, microtuble‐associated protein light chain 3B; *ATG 12*, autophagy‐related protein 12;TFEB, transcription factor EB; Con, control; St, starved.

Ten‐hour incubation of C2C12 cells in starvation medium significantly increased the mRNA expression of (P)RR, *ULK1*,* LC3B*, and *ATG12*, but not *TFEB* (Fig. 2A). Protein levels of full‐length (P)RR significantly increased with starvation (Fig. 2B). Nutrient starvation induced nuclear translocation of TFEB (Fig. 2C). Starvation increased the expression of autophagy marker, LC3B‐II (Fig. 2D).

### Knockdown of (P)RR or TFEB in starved C2C12 cells

**Figure 3:**
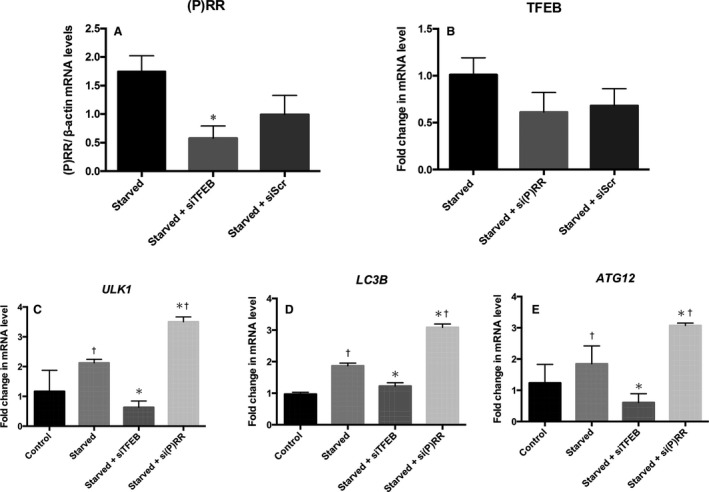
The mRNA expression of (P)RR in the untreated starved C2C12 cells and starved cells treated with siTFEB (*n* = 4) or scrambled siRNA (siScr, *n* = 4) (A). The mRNA expression of TFEB in the untreated C2C12 cells, si(P)RR‐treated cells (*n* = 4) and siScr‐treated cells (*n* = 4) (B). The mRNA expression of *ULK1* (C), *LC3B* (D), and *ATG12* (E) in the control C2C12 cells, starved cells, and the starved cells treated with siTFEB or si(P)RR (*n* = 4 in each). Data are expressed as mean ± SD. *P < 0.05 vs. starved cells, ^†^ *P* < 0.05 vs. control cells. (P)RR, (pro) renin receptor; TFEB, transcription factor EB; si, small interfering RNA oligonucleotide; Scr, scrambled siRNA.

**Figure 4:**
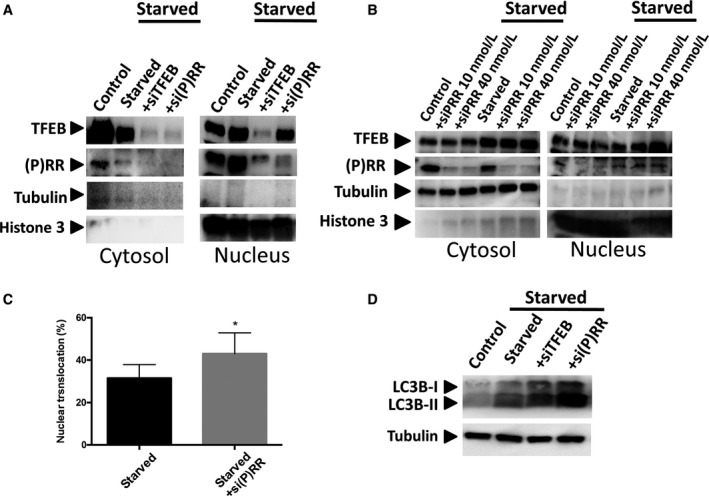
Representative western blots for TFEB and (P)RR in the cytosol and nucleus of control C2C12 cells, starved cells, and the starved cells treated with siTFEB or si(P)RR (A). Representative western blots for TEFB and (P)RR in the cytosol and nucleus of C2C12 cells starved with PBS for a few minutes showing an increase in nuclear TFEB with siPRR (B). Nuclear translocation of TFEB in the starved cells and the starved cells treated with si(P)RR (*n* = 6 in each) (C). Representative western blots for LC3B in control C2C12 cells, starved cells, and the starved cells treated with siTFEB or si(P)RR (D). Data are expressed as mean ± SD. *P < 0.05 vs. starved cells. si(P)RR, small interfereing (pro) renin receptor; siTFEB, small interfering transcription factor EB.

In the starved C2C12 cells, the transfection of 10 nmol/L siTFEB, which decreased TFEB mRNA by 60%, significantly reduced the expression of (P)RR mRNA (Fig. 3A). However, 40 nmol/L si(P)RR transfection, which decreased the (P)RR mRNA by 94%, did not significantly change the TFEB mRNA levels (Fig. 3B). In the starved cells, the mRNA expression of *ULK1*,* LCB3* and *ATG12* significantly decreased with siTFEB transfection and significantly increased with si(P)RR transfection (Fig. 3C–E). The western blot analysis showed that siTFEB transfection decreased the level of (P)RR in the nucleus of starved cells (Fig. 4A). Additionally, transfection of si(P)RR enhanced the nuclear translocation of TFEB in the starved cells (Fig. 4B and C). Treatment with si(P)RR augmented the increase in LC3B‐II by starvation (Fig. 4D).

### Effects of TFEB overexpression on (P)RR expression in starved C2C12 cells

**Figure 5:**
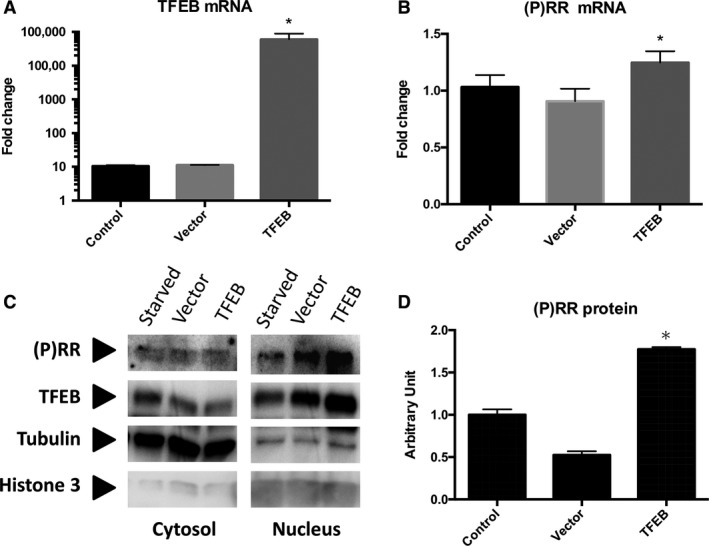
The mRNA expression of TFEB (A), (P)RR (B), representative western blots for TFEB and (P)RR (C), and the nuclear protein level of (P)RR (D) in control C2C12 cells (*n* = 4), vector‐transfected cells (*n* = 3), and TFEB‐transfected cells (*n* = 3). The cells were starved for 6 h in Figure 5C and D. TFEB, transcription factor EB; (P)RR, (pro) renin receptor.

The TFEB transfection significantly increased the TFEB mRNA level (Fig. 5A) and significantly elevated the mRNA (Fig. 5B) and protein levels (Fig. 5C and D) of (P)RR in C2C12 cells under starved condition.

## Discussion

In mice, nutrient starvation stimulated the expression of autophagy‐related genes and (P)RR in the skeletal muscles. Although the TFEB‐induced expression of (P)RR was involved in the regulation of autophagy‐related gene expression, (P)RR also inhibited the nuclear translocation of TFEB. These results suggest that (P)RR may act as a buffering mechanism to reduce excessive starvation‐induced autophagy.

The skeletal muscle is well known as a highly dynamic tissue, which promptly responds to physiological conditions and supplies energy needs. Autophagosome–lysosome system is the major proteolytic pathway in the muscle to recover the amino acids, which supply energy needs of the muscle cells (Maiuri et al. 2007; Levine and Kroemer 2008; Mizushima et al. 2008). Since the skeletal muscles are the largest reservoir of amino acids among all organs in the body, they play a pivotal role in the maintenance of amino acid balance by digesting their own proteins and organelles under low nutrient conditions (Mizushima et al. 2004). Therefore, autophagy in the skeletal muscles must be neither defective nor excessive, distinct from other tissues, and able to promptly adjust to the nutrient flux. This may explain why (P)RR is specifically overexpressed in the skeletal muscles during starvation.

The results indicated that starvation induced the nuclear translocation of TFEB and subsequent expression of autophagy‐related genes, consistent with the results of previous studies (Khambu et al. 2011; Settembre et al. 2011). In addition, the translocation of TFEB also resulted in the enhancement of (P)RR expression. Previous studies showed that (P)RR is essential for the autophagy–lysosome pathway (Kinouchi et al. 2010), suggesting that starvation‐induced upregulation of (P)RR may accelerate the autophagic pathway. In this study, however, the knockdown of (P)RR enhanced the starvation‐induced expression of autophagy‐related genes and nuclear translocation of TFEB. This result indicates that (P)RR may reduce the TFEB‐induced expression of autophagy‐related genes. The net effect of starvation on autophagy in the muscle cells is an increase as illustrated by the LC3B‐II results. Although the lack of (P)RR might cause dysfunction of autophagy (Kinouchi et al. 2010), the increase in (P)RR during starvation also has an inhibitory effect on the expression of autophagy‐related genes by suppressing the nuclear translocation of TFEB. Appropriate expression of (P)RR seems to be essential for proper regulation of autophagy flux, which is fundamental for the homeostasis of skeletal muscles (Grumati and Bonaldo 2012).

In skeletal muscles of fasted mice, the protein expression of s(P)RR but not (P)RR was elevated, although the mRNA level of (P)RR was significantly increased. The enhanced expression of furin, a processing enzyme of (P)RR, is considered to be responsible for the increased levels of s(P)RR but not (P)RR in the fasted skeletal muscles. The (P)RR protein before cleavage by furin, the C‐terminal fragment of (P)RR, and/or the s(P)RR may contribute to the autophagic pathway necessary for the homeostasis of skeletal muscles. The muscle and myoblast cells may not express significant amount of renin or prorenin, but the involvement of (P)RR in the regulation of autophagy may be ligand‐independent (Ichihara and Kinouchi 2011). In this respect, the C‐terminal fragment of (P)RR harboring the binding site for v‐ATPase may be the most functional component in autophagy regulation, and s(P)RR may be assessed as a surrogate marker reflecting the action of the C‐terminal fragment.

We recently showed that serum s(P)RR levels are elevated in patients with low body mass index (BMI) and Graves’ disease (GD) compared to those in patients with high BMI, and their serum s(P)RR levels normalized with medical treatment (Mizuguchi et al. 2016). On the contrary, serum s(P)RR levels in patients with hypothyroidism were similar to those in patients with euthyroidism, independent of BMI. These findings suggest that s(P)RR may increase with hyperthyroidism‐induced starvation rather than by the direct effects of thyroid hormone. This study may also explain why serum s(P)RR levels are elevated in the lean patients with GD. The starvation caused by hyperthyroidism can enhance the expression of (P)RR in the skeletal muscles of patients with GD and subsequently increase serum s(P)RR levels. Thus, the measurement of serum s(P)RR level may be useful for the evaluation of nutrient condition at least in patients with GD.

In conclusion, starvation induces the nuclear translocation of TFEB, which stimulates the expression of autophagy‐related genes, leading to the enhancement of autophagy. This mechanism is essential for the homeostasis of cells, in particular the skeletal muscle cells, since the skeletal muscles are one of the most important tissues accounting for the regulation of metabolism. In the skeletal muscles, therefore, proper control of autophagy flux is needed. This study showed that (P)RR plays a buffering role in the starvation‐induced autophagy by affecting the nuclear translocation of TFEB. (P)RR, which increases during starvation, may be one of the important factors to control the autophagy flux properly in the skeletal muscles.

## Conflicts of Interest

None.
